# Neutrophil proteins as potential biomarkers for a sputum-based tuberculosis screening test

**DOI:** 10.3389/fimmu.2025.1636909

**Published:** 2025-10-20

**Authors:** Mark Chambers, Farina Karim, Matilda Mazibuko, Zoey Mhlane, Lindiwe Madziwa, Yunus Moosa, Sashen Moodley, Monjurul Hoque, Emily Beth Wong, Andriette Hiemstra, Stephanus Theron Malherbe, Belinda Kriel, Kim Stanley, Ilana Claudia van Rensburg, Ayanda Shabangu, Bronwyn Smith, Gerhard Walzl, Nelita Du Plessis, Timothy R Sterling, Mark Hatherill, Alasdair Leslie

**Affiliations:** ^1^ Africa Health Research Institute, Department of Basic and Translational Science, Durban, South Africa; ^2^ School of Laboratory Medicine and Medical Sciences, University of KwaZulu-Natal, Durban, South Africa; ^3^ Department of Infectious Diseases, Nelson R. Mandela School of Clinical Medicine, University of KwaZulu-Natal, Durban, South Africa; ^4^ Wits Reproductive Health and HIV Institute, University of the Witwatersrand, Johannesburg, South Africa; ^5^ Division of Infectious Diseases, Massachusetts General Hospital, Boston, MA,, United States; ^6^ South African Medical Research Council Centre for Tuberculosis Research, Biomedical Research Institute, Division of Immunology, Faculty of Medicine and Health Sciences, Stellenbosch University, Cape Town, South Africa; ^7^ Division of Infectious Diseases, Department of Medicine, Vanderbilt University School of Medicine, Nashville, TN, United States; ^8^ South African Tuberculosis Vaccine Initiative, Institute of Infectious Disease & Molecular Medicine and Division of Immunology, Department of Pathology, University of Cape Town, Cape Town, South Africa; ^9^ Department of Infection and Immunity, University College London, London, United Kingdom

**Keywords:** tuberculosis, neutrophil proteins, screening test, PDL1, ELISA -enzyme-linked immunosorbent assay

## Abstract

**Introduction:**

The development of a rapid and affordable assay to screen participants for Q12 additional testing could streamline TB screening in resource-limited settings and for community-wide health screens. Sputum remains the primary testing sample, making it potentially ideal for a screening testing. Neutrophils are highly expanded in sputum from individuals with pulmonary TB with high specificity and have potential as a biomarker for TB.

**Methods:**

Three neutrophil associated proteins, neutrophil gelatinase associated-lipocalin (NGAL), the protein heterodimer S100A8/A9 and the protein death ligand-1 (PDL-1), were measured in presumptive TB cases from participants attending a primary healthcare clinic in Durban, South Africa, using commercially available ELISAs on a total of 79 participants from a 109-participant cohort. Participants with microbiologically confirmed TB were sampled after 1 month of treatment. Proteins were also measured in tongue swab samples in participants from this cohort at baseline. Baseline results were confirmed in a second TB cohort which recruited a total of 51 participants with presumptive TB from the Western Cape. Finally, we investigate sputum neutrophil protein levels in individuals with community diagnosed asymptomatic TB.

**Results and discussion:**

Significant increases in all proteins were detectable in sputum from clinic-diagnosed TB participants relative to symptomatic controls. Performance approached the WHO target product profile for a TB triage test, with ROC AUCs reaching 0.866 (with a 95% confidence interval of 0.7683 – 0.9633) in the case of S100A8/A9. Sputum protein levels did not correlate with bacterial burden and did not consistently decrease following one month of drug therapy. Only PDL-1 was detectable in mouth swab samples. Sputum neutrophil proteins tended to be elevated in participants with asymptomatic community diagnosed TB, as compared to asymptomatic community controls within the Vukuzazi cohort using a sample size of 42 participants, although this was not significant. This study provides a proof of principle that neutrophil proteins can be easily measured in standard sputum samples and have potential as a screening test for TB. However, more work is needed to explore whether this approach, using these three neutrophil proteins, can meet the WHO target product profile for a triage test worth developing further.

## 1 Introduction

Tuberculosis (TB) remains the leading cause of death from infectious disease globally, disproportionately affecting regions with limited health resources (1). Most TB testing is triggered by the onset of TB symptoms, but even in high burden settings the yield of TB cases via symptomatic screening is low (2). TB diagnosis ideally involves the detection of the causative agent, *Mycobacterium tuberculosis* (Mtb), via culture, or the presence of Mtb DNA, most commonly via GeneXpert (3–5), which can be time consuming and expensive (6). A screening test for people with suspected TB could help streamline testing of those at highest risk of TB disease and prompt additional testing to rule out TB. Standard TB tests generally use sputum as their substrate and rely on the presence of sufficient bacteria or nucleic acid in the sample. This introduces an additional problem as the positivity of a sputum sample can be sporadic, as illustrated by testing or pooling two separate samples; this significantly increases TB yield (7). Consequently, many TB programs recommend testing two or three sputum samples to rule out TB. Furthermore, sputum samples from certain groups, such as children and individuals with advanced HIV, tend to be paucibacillary (8). Because sputum remains the principal sample for TB testing, a screening test that utilizes sputum would have the added advantage of requiring no additional sampling as the same sputum sample could be used for multiple tests.

In addition to symptomatic or clinical TB, it is becoming increasingly apparent that a large proportion of prevalent TB does not show classic signs of TB, up to 80% in some settings (9). Consequently, community-wide, symptom agnostic TB testing is suggested as an effective way to tackle the TB pandemic in high burden settings (10). However, community-wide screening, potentially on an annual basis, is expensive and not feasible. Again, a screening test that streamlines downstream testing to those most at risk of TB disease could significantly reduce these challenges. The World Health Organization recognises the value of a TB triage test and has designated the target product profile as not less than 90% sensitivity and 70% specificity (11).

Neutrophilia is a hallmark of TB disease and neutrophils are one of the first immune responders to Mtb infection in the lung (12–14). We have previously shown that neutrophils and neutrophil-associated proteins are highly enriched in the sputum of individuals with microbiologically-confirmed pulmonary TB compared to healthy controls and symptomatic individuals attending a TB clinic subsequently determined to not have active TB (15). Importantly, this association was not affected by concurrent HIV infection.

The aim of this study was, therefore, to assess the detectability of 3 neutrophil associated proteins (PDL-1, N-GAL and S100A8/9) in the sputum of participants with microbiologically confirmed TB by culture and/or GeneXpert, utilizing minimal sample processing. While PDL-1 is not exclusively expressed in neutrophils, prior work using flow cytometry showed increased expression in myeloid cells in TB granulomas and circulation (16, 17), in addition to our own work showing it was highly upregulated in sputum neutrophils during active TB. Thus, PDL-1 may be representative of a component of TB derived neutrophil activation. We reasoned this could provide pilot data towards an inexpensive, clinically relevant rapid diagnostic test (RDT) as a triage for further testing by GeneXpert or culture. Data were compared to symptomatic participants attending the same TB clinics but not receiving a TB diagnosis or with a diagnosis other than TB. In addition, we investigated the ability to detect PDL-1, N-GAL and S100A8/9 in tongue swabs, an alternative non-sputum based method of detecting Mtb DNA that is showing promise (18). We subsequently compared these tongue swabs with sputum samples from the same participants to determine efficacy. Finally, we investigated the level of PDL-1, N-GAL and S100A8/9 in sputum from community detected asymptomatic TB cases and asymptomatic controls to assess the potential of this approach as a screening test for symptom-agnostic TB.

## 2 Materials and methods

### 2.1 Cohort descriptions

A total of 109 study participants over the age of 18, 64 males and 35 females, were recruited at the KwaDabeka clinic under the AHRI RePORT South Africa (SA) TB cohort study after presenting with symptoms of TB and were tested using GeneXpert and confirmed by liquid culture (BD Bactec MIGIT). No patients had any previous history of TB. All participants provided written informed consent for sample collection. Tongue swabs were also obtained in a subset of participants. The study protocol, data collection tools and associated consent forms were approved by the University of KwaZulu-Natal biomedical research Ethics Committee (BE285/16).

A second cohort, the SUN RePORT SA TB cohort, coordinated by Stellenbosch University, recruited 51 participants from several healthcare facilities in the Western Cape province of South Africa. For the community cases from the SUN RePORT SA TB cohort, 31 sputum samples were collected from household contacts of microbiologically confirmed TB, these potential asymptomatic cases were subsequently screened for TB. Ethical approval for this study was obtained through the Stellenbosch Human ethics (SUN HREC: B20/10/006; BIOMARKER APPROACHES FOR SYMPTOMATIC, SUBCLINICAL AND INCIPIENT TUBERCULOSIS (RePORT II SA) and Environmental and biosafety ethics (SUN RECBES: BES-2023-28067; Biomarker Approaches for symptomatic, subclinical and incipent tuberculosis).

For the Vukuzazi cohort, the eligible study population consisted of all resident members of the southern population intervention platform (PIP) area who were aged ≥15 years during the baseline data collection (May 2018–March 2020) which was initially defined as the Africa Centre Demographic Information System (ACDIS) cohort. The PIP encompasses a surveillance area of approximately 845 km², monitoring approximately 140,000 individuals across more than 20,000 households in rural KwaZulu-Natal, South Africa (19). Non-resident members were excluded from participation. Ethical approval for the study was obtained from the Ethics Committees of the University of KwaZulu-Natal (BE560/17), London School of Hygiene & Tropical Medicine (#14722), the Partners Institutional Review Board (2018P001802), and from the University of Alabama at Birmingham (#300007237). A 42-participant subset of this population, with 35 community diagnosed TB cases alongside controls matched for sex, age and HIV status, was utilized for this study, under double-blinded experimental conditions.

### 2.2 Sputum collection

Sputum samples collected in Durban were induced using saline solution under supervision, samples were transported on ice to the lab and processed rapidly (within 1 hour) under Biosafety level 3 conditions. Sputum samples were mixed 1:2 (v/v) in N-acetyl cysteine (NAC, ThermoFisher Scientific) and agitated for 10 minutes at room temperature. Sputum/NAC were mixed and subsequently stored at -80 °C.

In the case of the SUN RePORT SA TB cohort, freshly collected sputum was digested and homogenized using a 1,4-Dithiothreitol (DTT) solution. Briefly, raw sputum was diluted 1/10 with 0,1% DTT and vortexed for 20 seconds, followed by mechanical shaking at room temperature for 20 minutes at 60 rpm. Immediately thereafter, aliquots of sputum were stored at -80˚C and were later shipped on dry ice for analysis. The RePORT SA SUN cohort sputum samples were then further processed and stored as above at -80 °C. Mouth swabs were collected utilizing regular Nylon COPAN Floqswabs (HCPN552C). Mouth swabs were submerged in lysis buffer containing 1% Triton X-100 (v/v) in PBS and store at -80 °C. Lysed samples were then thawed and run on ELISAs as per manufacturer’s instructions.

In the case of sputum samples obtained from the Vukuzazi cohort, samples were frozen at -80 ˚C upon retrieval. These samples were then thawed at room temperature and subsequently processed with NAC as before.

### 2.3 Analysis of neutrophil protein levels in sputum and plasma

Neutrophil associated proteins were quantified by Enzyme-linked immunosorbent assays (ELISA) for sputum. Sputum was processed as above, thawed at room temperature, and then prepared for ELISA as per manufacturer’s instructions. ELISAs for NGAL (HK330), PDL-1 (HK324) and S100A8/A9 were purchased from R&D systems (DS8900). All ELISAs were run as per manufacturer’s instructions alongside standard protein curves. The dilution factors used were 1 in 2000, 1 in 5 and 1 in 20000 for NGAL, PDL-1 and S100A8/A9 respectively.

### 2.4 Statistical analysis

All statistical analyses were carried out in Graphpad Prism using one way ANOVA to compare groups with Dunnett’s correction for multiple comparison.

## 3 Results

**Figure 1:**
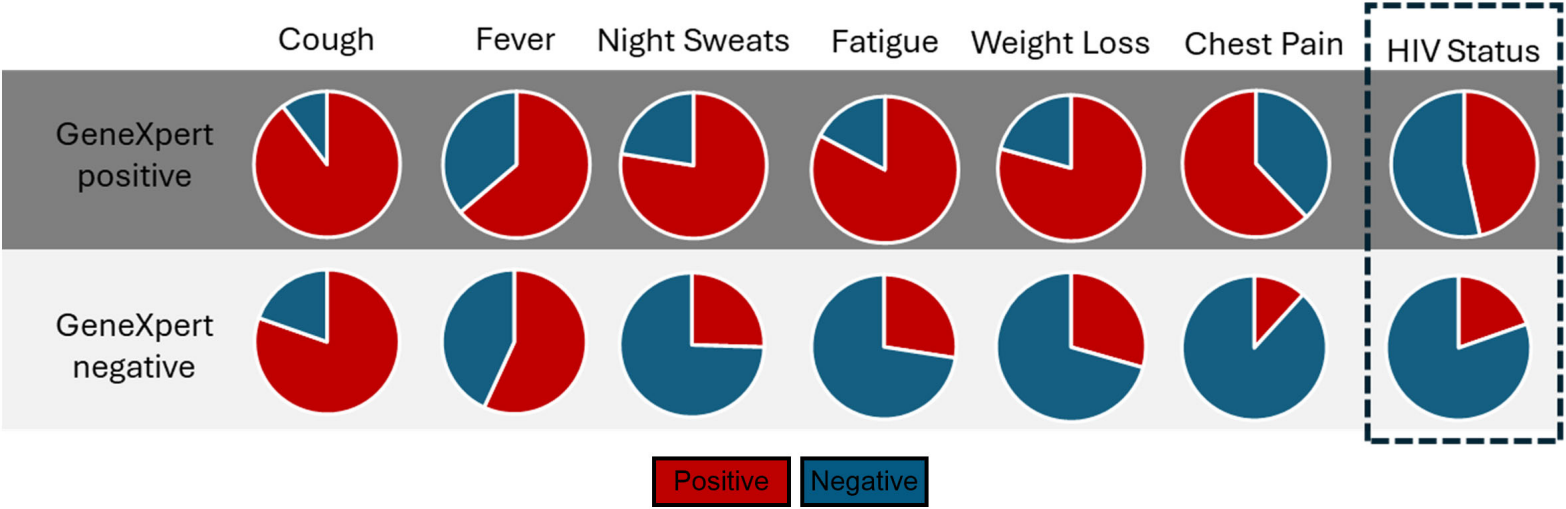
The distribution of symptoms and HIV status of participants recruited from the AHRI RePORT SA TB cohort. All patient symptoms recorded at time of enrolment, alongside HIV status. All enrolled participants were symptomatic clinic attendees.

Initially, we tested sputum samples obtained from the AHRI RePORT SA TB cohort. All participants were tested by GeneXpert and are grouped as GeneXpert positive vs. negative. All participants displayed at least one classical TB symptom, either persistent cough, fever, unintended weight loss, night sweats, fatigue and chest pain, at the time of testing (summarized in **Figure 1**). Of the GeneXpert-negative participants, 19.6% were HIV-positive compared to the 46.6% HIV-positive GeneXpert positive participants, consistent with the increased risk of TB disease associated with HIV infection. These data were consistent with the high prevalence of HIV in KZN, which was reported at 19.6% in 2024 by the Department of Health (20) and the high HIV/TB coinfection rate of 59% reported in the 2024 South African National TB Survey.

To quantify neutrophils in sputum, we selected 3 abundant neutrophil-associated proteins for which commercial ELISAs were available and should have been detectable in sputum. Neutrophil Gelatinase (NGAL) is a constitutively expressed neutrophil protein, although it can also be expressed in airway epithelial cells; it is detectable in sputum samples of individuals with chronic obstructive pulmonary disease (COPD) (21). S100A8/9 is a highly expressed neutrophil protein that is elevated in the plasma of individuals with active TB (22), and in the sputum of individuals with COPD and severe COVID (23). Finally, PDL-1 was selected because we had observed a highly significant upregulation of PDL-1 in sputum neutrophils of people with TB disease (15).

**Figure 2:**
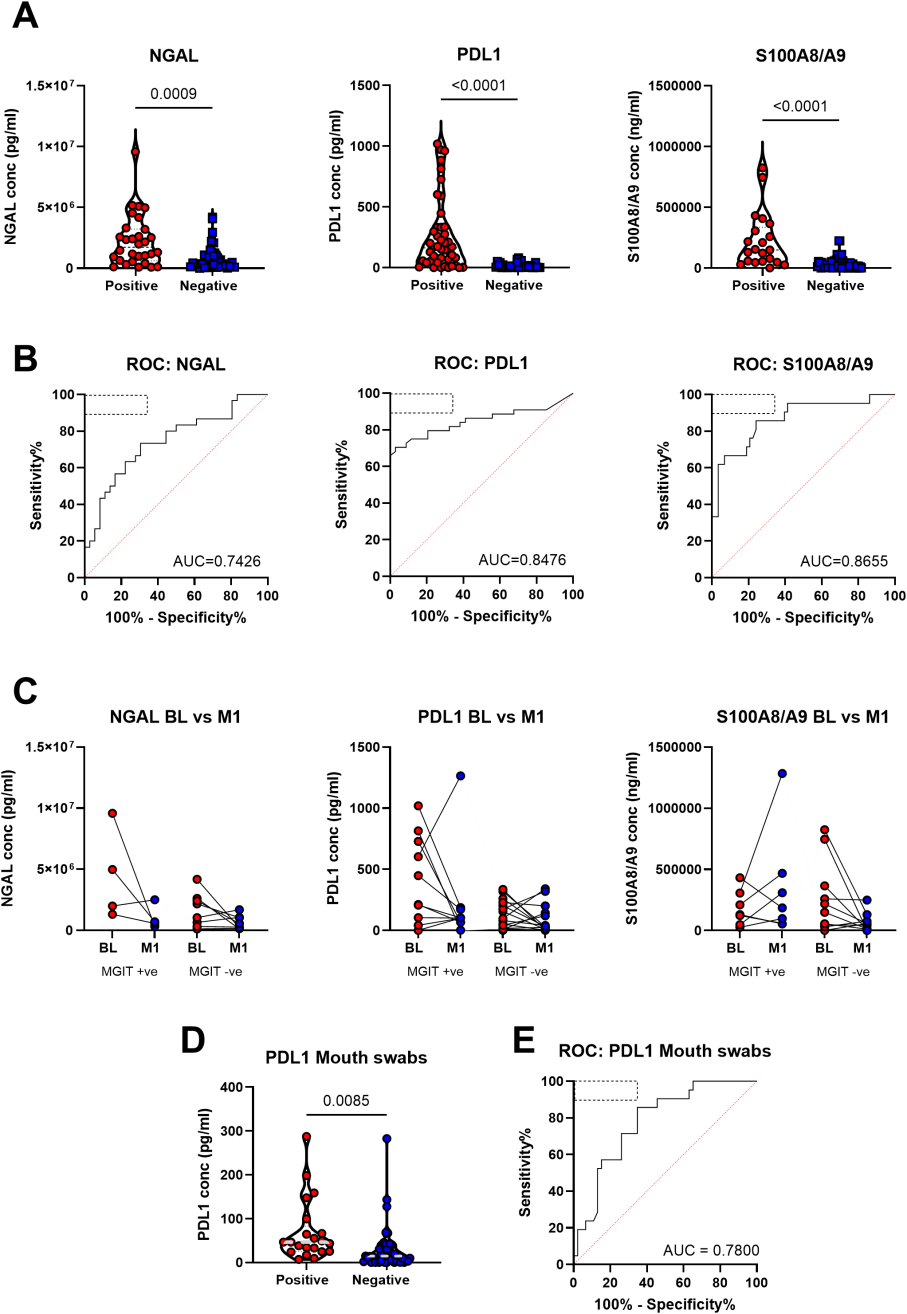
Neutrophil-related proteins in patient sputum were significantly associated with tuberculosis disease state in the AHRI RePORT SA TB cohort. **(A)** The levels of the neutrophil associated proteins neutrophil gelatinase-associated lipocalin (NGAL), programmed death-ligand 1 and the S100A8/A9 heterodimer are shown as a product of GeneXpert result. **(B)** The receiver operator curves for each protein are demonstrated with the WHO target product profile indicated by the dashed box with area under curves being reported. **(C)** Protein levels at baseline and month 1 after treatment were recorded and reported based off clearance of the TB at the month 1 timepoint, with all patients being GeneXpert and MGIT positive at baseline. **(D)** Baseline sputum and mouth swab PDL-1 levels comparing TB positive and TB negative. **(E)** The receiver operator curve for the mouth swab results is demonstrated with the WHO target product profile indicated by the dashed box with the area under the curve reported. A total of 79 sputum samples were collected, with confidence intervals of 95% being indicated for area under curve values.

Consistent with our previous observation that neutrophils were highly upregulated in sputum, NGAL, S100A8/9 and PDL-1 were all significantly elevated in sputum samples of participants with symptomatic TB compared to symptomatic non-TB controls within the AHRI RePORT SA TB cohort (**Figure 2A**). To test the potential diagnostic utility of this test, we performed a Receiver Operator Curve (ROC) analysis for each protein, generating an Area Under the Curve (AUC) of 0.74 (95% CI = 0.62-0.86), 0.85 (95% CI = 0.76-0.94) and 0.87 (95% CI = 0.77-0.96) for NGAL, PDL-1 and S100A8/9 respectively (**Figure 2B**). The relatively poor performance of NGAL was due to elevated levels in a proportion of Non-TB controls. Consequently, NGAL exhibited a reduced sensitivity and specificity of 64 and 71% respectively. S100A8/A9 demonstrated a sensitivity and specificity of 81% and 76% and PDL-1 a sensitivity and specificity of 83% and 88% respectively, thus approaching the target product profile (TPP) for a TB triage test (**Figure 2B**), TPP indicated by hatched oblong box in the ROC curve). In addition, these differences were still apparent in HIV-positive individuals, although the sample size was reduced (**Supplementary Figure S1**).

We next tested whether sputum neutrophil protein level correlated with TB severity, using sputum bacterial load as a proxy. For this, participants were grouped based on GeneXpert readings as having high, medium or low bacterial burden as determined using cycle threshold. This revealed no significant difference in protein level between TB positive groups (**Supplementary Figure S2**). Although somewhat anecdotal, three participants were subsequently found to be culture negative, despite a positive GeneXpert test, which could indicate previous TB diagnosis resulting in false positive GeneXpert results, and all three had very low or undetectable levels of sputum NGAL, PDL-1 and S100A8/9 (**Supplementary Figure S3**). However, this number is too small for meaningful statistical testing. As an alternative approach, we correlated sputum neutrophil protein level with sputum bacterial burden based on MIGIT culture conversion [Time To Detection (TTD)], where low TTD equates to high bacterial burden). Again, this suggested no association between sputum bacterial load and sputum neutrophil protein level (**Supplementary Figure S4**).

To determine the value of measuring sputum neutrophil proteins in monitoring TB treatment response, additional sputum samples were taken after 1 month of standard TB therapy in a subset of individuals with confirmed drug-susceptible TB. Participants were divided into groups according to whether sputum samples were culture positive or negative at one month (**Figure 2C**). Although we observed a marked decline in sputum neutrophil proteins in some participants, particularly those with high abundance at base line, overall, we did not detect significant differences across all three biomarkers. This was true regardless of culture-positivity at month 1, potentially consistent with a lack of correlation between MGIT time to detection and neutrophil protein levels (**Supplementary Figure S4**). Again, although anecdotal, one participant displayed a large increase in the level of S100A8/9 and PDL-1 at month 1. Although meeting the inclusion criteria of drug-susceptible TB this individual was subsequently found to have a greatly elevated bacterial burden at month one exhibiting a MGIT TTD of 4 days. Comparison of protein levels between male and female revealed no significant differences (**Supplementary Figure S5**). In addition, no correlations were observed between protein level participant age (**Supplementary Figure S6**).

A subset of the above participants also provided mouth swab samples. These were tested to test the viability of detecting these neutrophil protein biomarkers in patient saliva (**Supplementary Figure S7**). However, only PDL-1 was detectable using mouth swabs. Nevertheless, PDL-1 levels were significantly elevated in GeneXpert positive participants compared to GeneXpert negative (**Figure 2D**) and in ROC curve analysis had an AUC of 0.78 (95% CI = 0.67-0.89) and a sensitivity and specificity of 71% and 74% respectively (**Figure 2E**).

**Figure 3:**
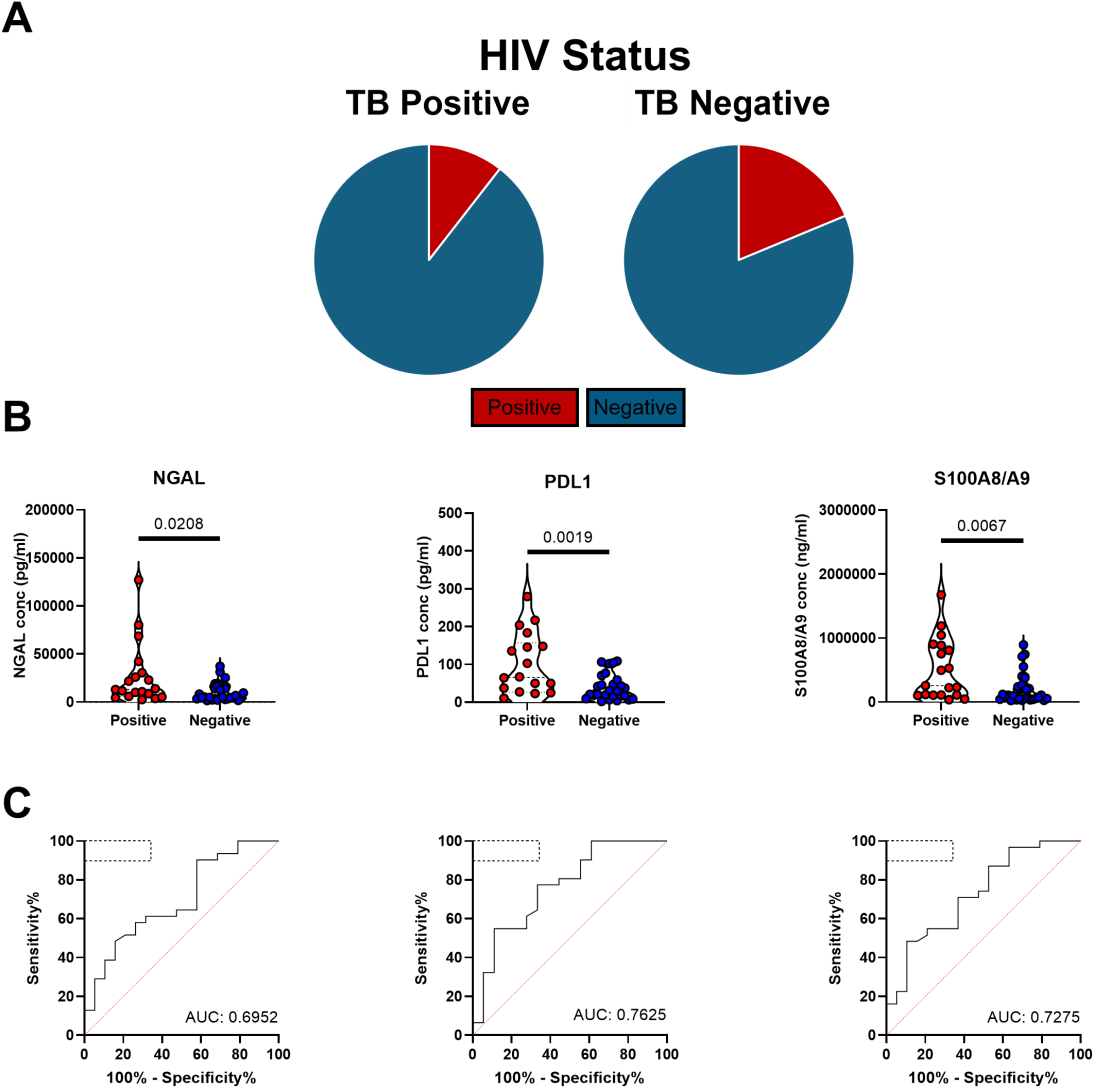
Neutrophil proteins remain significantly increased in TB positive patients in the SUN RePORT SA TB cohort with reduced HIV burden. **(A)** HIV burden in both TB positive and negative patients in the Stellenbosch cohort. **(B)** The levels of the neutrophil associated proteins neutrophil gelatinase-associated lipocalin (NGAL), programmed death-ligand 1 and the S100A8/A9 heterodimer are shown as a product of GeneXpert result. **(C)** The resulting receiver operator curves for each protein with area under curves being reported with the WHO target product profile indicated by the dashed box with the area under the curve reported. A total sample size of 51 samples was used, with confidence intervals of 95% being indicated for area under curve values.

Having established the diagnostic potential of measuring sputum neutrophil proteins, we next turned to a second cohort to confirm these findings and test the robustness of the approach. For this, sputum samples were obtained from the SUN RePORT SA TB cohort, collected from similar participants under the RePORT SA common protocol. However, sputum samples were processed differently, as described in the methods section, using DTT as opposed to NAC to disassociated mucins prior to freezing. In addition, there was a lower incidence of HIV as compared to the KwaZulu-Natal cohort (**Figure 3A**). Overall, of the GeneXpert positive participants, 10.5% were HIV positive while 18.8% of GeneXpert negative participants were. Again, we observed significantly higher levels of all three neutrophil proteins in the sputum of participants with microbiologically confirmed TB compared to TB negative controls (**Figure 3B**). However, the differences were less striking than initially observed. Consequently, we observed poorer performance in ROC curve analysis, with PDL-1 having a sensitivity and specificity of 71% and 67%, NGAL 61% and 63% and S100A8/A9 63%, and of 71% respectively (**Figure 3C**). These sensitivities and specificities were markedly decreased in comparison to the AHRI RePORT SA TB cohort. To determine if this might be the result of the sample processing methodology, comparisons in protein abundance as detected by ELISA were made between all three sites (**Supplementary Figure S8**). These data showed DTT processing to yield higher protein levels overall, suggesting protein yield is unlikely to explain the lower assay performance. A head-to-head comparison of potential sputum processing methods will be required to identify the optimal approach.

**Figure 4:**
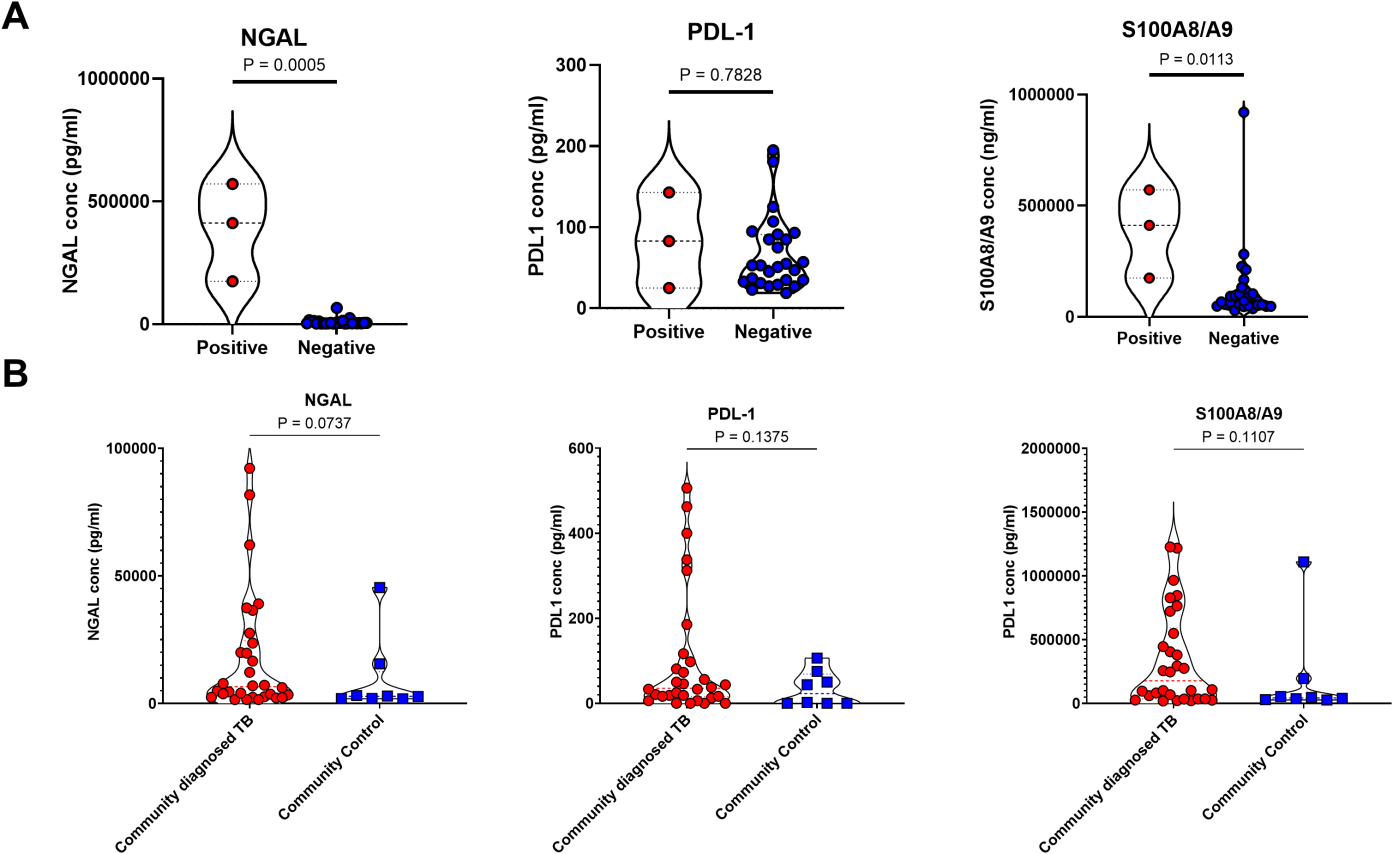
Sputum neutrophil proteins are not consistent enough for classification of subclinical tuberculosis in the SUN RePORT SA TB cohort or the Vukuzazi cohort. **(A)** The levels of the neutrophil associated proteins neutrophil gelatinase-associated lipocalin (NGAL), programmed death-ligand 1 and the S100A8/A9 heterodimer are shown as a product of GeneXpert result. **(B)** The levels of NGAL, PDL-1 and S100A8/A9 also measured in the Vukuzazi cohort, comparing the community diagnosed TB and the community controls. A total sample size of 31 samples was used in the SUN RePORT SA TB cohort. A total sample size of 42 samples was used in the Vukuzazi cohort.

Finally, we sought to test the utility of this approach for detecting asymptomatic TB. For this we made use of two separate cohorts. First, stored sputum samples, processed using DTT (as above), were obtained from the SUN RePORT SA household contact survey, in which sputum samples were obtained from asymptomatic household contacts of confirmed TB cases. A fraction of these participants was found to have microbiologically confirmed TB, consistent with the high prevalence of asymptomatic TB in the community. These samples were run in a blinded fashion and then data analysed according to microbiological status. Unfortunately, only three samples with microbiologically confirmed TB were available for testing, preventing meaningful statistical analysis. However, these individuals had higher levels of NGAL and S100A8/9 compared to microbiologically negative household contacts (**Figure 4A**).

To test these observations further, we turned to stored samples from the Vukuzazi community health screening study which identified prevalent TB and collected biosamples from a community based in rural KwaZulu Natal between May 2018 – March 2020 (9). In keeping with other national and international TB screening programmes, this study found the majority (80%) of community diagnosed TB, based on GeneXpert or chest X-ray findings, were asymptomatic. Asymptomatic sputum samples were compared to asymptomatic community controls obtained during the same survey with no evidence of TB disease. Of note, sputum samples from this cohort were stored without processing, which may have affected sample quality. However, all three neutrophil proteins were detectable in sputum and showed a trend for increased levels in asymptomatic TB cases compared to community controls, although this was not statistically significant (**Figure 4B**), and was not affected by method of TB diagnosis (not shown).

## 4 Discussion

We investigated the potential of measuring sputum neutrophil proteins as a pre- screening test prior to expensive and time-consuming TB testing. Such a test could be useful in several settings, such as facility based screening of individuals attending primary health care clinics (24), or community-based screening in high burden settings. Crucially, even in high burden regions of South Africa, the probability of prevalent TB remains extremely low, approximately 1% in the community (9). In this context, a low-cost rapid tool to synergize with GeneXpert testing could reduce the diagnostic burden on primary care facilities and overall cost. Modelling suggests that up to 75% of negative GeneXpert tests could be prevented in the presence of a sufficiently sensitive and specific rule-out test (25). Estimates from Uganda suggest that triaging testing in health care facilities with an estimated prevalence of 5% remained cost effective using relatively expensive methods such as digital chest x-ray or serum Crp (costing approximately 25 USD). Lateral flow assays are being explored to quantify serum TB biomarkers (typically cytokines) and can typically be delivered at a much lower cost (26). However, more work is needed to establish the potential of this approach further and determine use-case scenarios.

Overall, we demonstrated that the neutrophil associated proteins NGAL, PDL-1 and S100A8/9 were all detectable in stored sputum samples with minimal processing using commercially available ELISAs. All three proteins were significantly elevated in symptomatic TB cases compared to symptomatic TB negative controls attending the same TB clinic, thus representing a potential target population for a TB screening testing. The sensitivity and specificity of sputum (81% and 76%) and S100A8/9 (83% and 88%) approached the TPP for a WHO TB triage test of 90% sensitivity and 70% specificity. Crucially these sensitivity and specificity values were recorded despite the high level of HIV prevalence within the AHRI RePORT SA TB cohort. We replicated these findings in a separate cohort.

Surprisingly, we found no association between sputum bacterial load and neutrophil protein abundance. In addition, we found variability in the extent to which neutrophil proteins declined during the first month of TB treatment, irrespective of sputum culture conversion. It is possible that neutrophil levels in sputum are associated with levels of inflammation in the lung, which may not correlate closely with Mtb burden. However, more work is required to test this association.

While no correlation between neutrophil protein levels and Mtb level was observed, it is important to note that bacterial load often only correlates weakly with clinical markers of disease severity such as systemic inflammation at baseline (27, 28). Furthermore, it is likely that the limited sample size available in this study reduced our power to detect weaker correlations between these metrics. Nevertheless, Neutrophil proteins are known to be elevated in tissue inflammation and injury and thus may be of some use as indicators of patients at higher risk of disease progression while on treatment. The caveat being that variability in disease state might make such measurements less straightforward and thus further work should be carried out.

We focused on sputum samples with the rationale that sputum remains the main biosample for diagnosing TB and could therefore be co-opted for screening without the need for additional training or resources. However, we also explored the possibility of using mouth swabs to detect TB associated spikes in neutrophil proteins, as this is being explored as an alternative sampling technique for TB testing (23). In a limited sample size only PDL-1 was detectable using mouth swabs. PDL-1 is known to be upregulated in the oral mucosa during inflammatory conditions (29), potentially via epithelial cells, which are known to express PDL-1 in response to local inflammation in the oral mucosa (30). However, unlike PDL-1, NGAL and S100A8/A9 are expressed almost exclusively by neutrophils (31, 32). Therefore, we interpret the lack of these proteins in tongue swabs to indicate a low abundance of neutrophils in this sample type. Whether PDL-1 expression in the oral mucosa, irrespective of cellular source, offers a potential alternative biomarker is unclear from these data, but maybe limited due other conditions that may lead to inflammation at this site.

It is worth noting there are several limitations to this study, first of which is the differences in processing across the three cohorts. Despite these processing differences however, significant differences were still detectable across the three cohorts, though the changes in ROC AUCs, indicates the potential role processing may play in optimizing this assay for point of care testing. Secondly, for the AHRI RePORT SA cohort, initial ELISA testing was not carried out on identical sample lists between the three protein tests, slightly reducing the ability to compare across the three neutrophil protein tests, however as this is an exploratory study into the diagnostic potential of neutrophil proteins this effect is minimal.

Finally, we explored the potential utility of this approach in detecting asymptomatic TB in community. Unfortunately, with limited sample number and differing sputum processing approaches it is not clear at present if this approach has any merit. Although we attempted to minimise sample processing, in line with developing a simple test, our observations suggest that small differences in processing approach may impact assay performance. Ultimately, although ELISAs were used here, we envision such a test could be translated into a simple dip stick test. However, more work is needed to confirm these findings and optimise sample collection and processing to justify further development. In addition, alterative sputum proteins may have a better diagnostic yield, either alone or in combination. Despite these caveats, however, we believe our findings suggest that parallel testing of sputum samples for a TB biomarker could have significant value as a TB triage or screening test.

The goal of developing a field deployable rapid diagnostic test capable of hitting the WHO targets for a triage test is important, particularly in low-middle income countries. Therefore, further work into development of a lateral flow assay could be carried out as this is the clearest direction to achieve this goal while still being relevant in high burden, resource-limited settings. Overall, this study’s findings support further investment into neutrophil-based biomarkers and their relevance in scalable diagnostics. These tests would serve as a useful complement to existing TB tests and could help lessen the burden on primary health care facilities that rely on GeneXpert and MGIT testing.

## Data Availability

The raw data supporting the conclusions of this article will be made available by the authors, without undue reservation.
