# Supplementary figures and images for: Neutrophil proteins as potential biomarkers for a sputum-based tuberculosis screening test
Source: Front Immunol. 2025 Oct 20;16:1636909. doi: 10.3389/fimmu.2025.1636909 (PMC12580308; doi:10.3389/fimmu.2025.1636909)

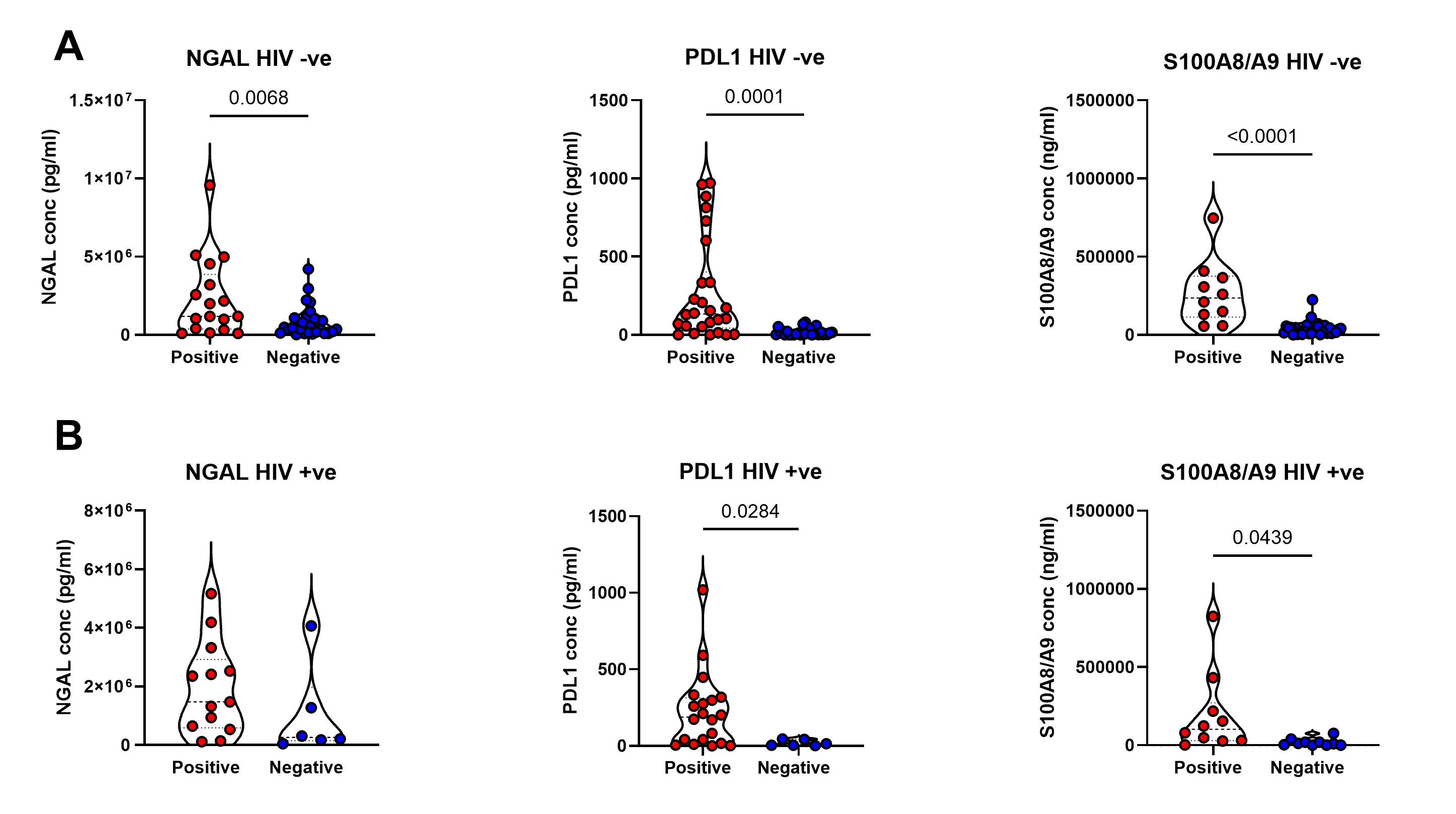

Supplement: Supplementary file 1 [file Image1.tif]

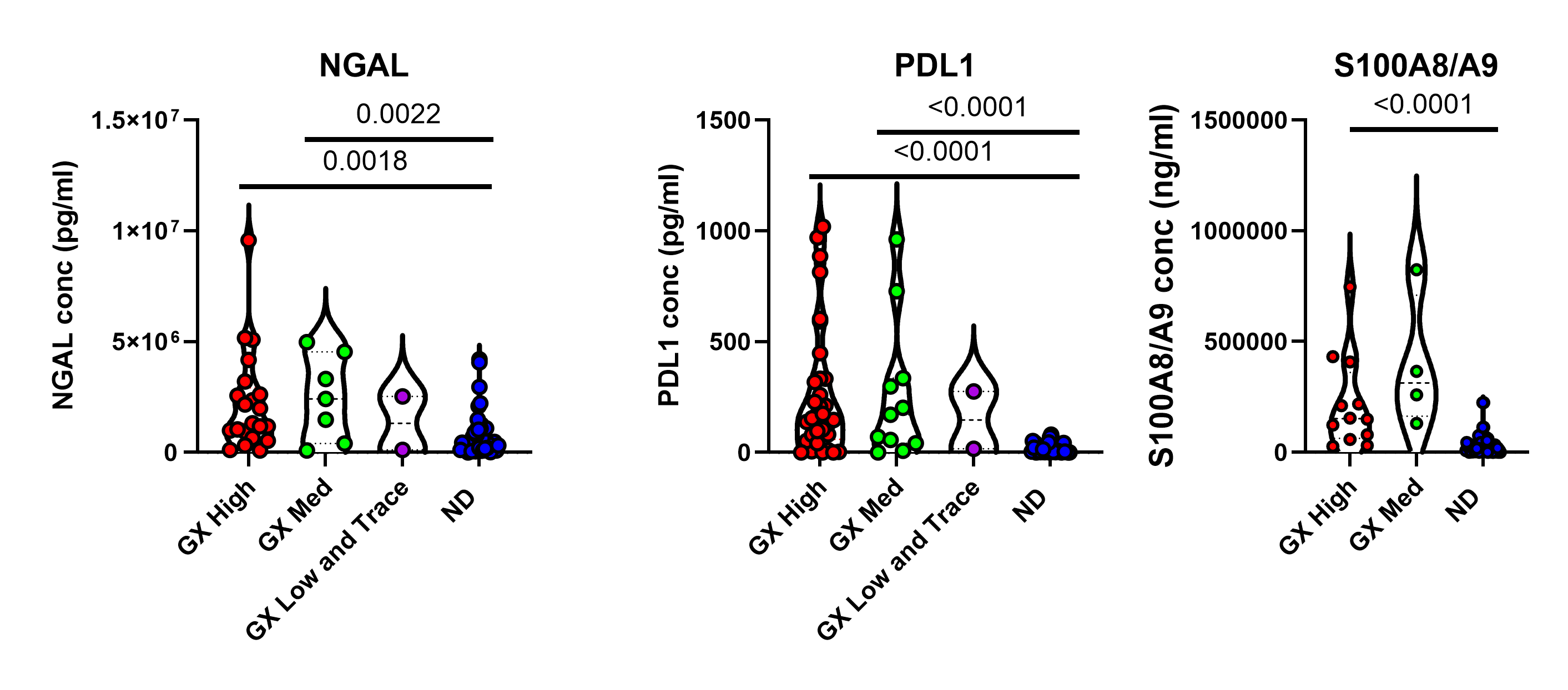

Supplement: Supplementary file 2 [file Image2.tif]

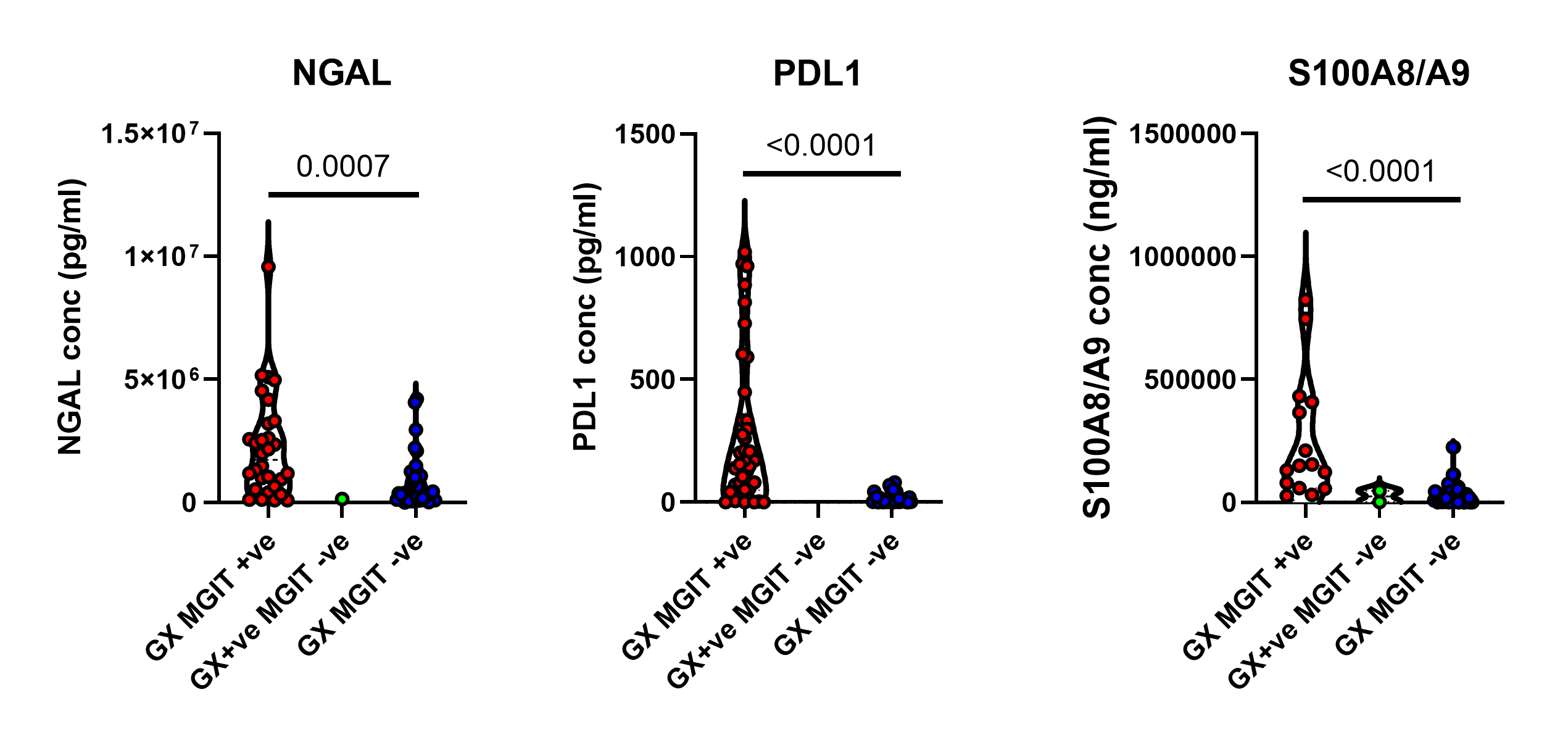

Supplement: Supplementary file 3 [file Image3.tif]

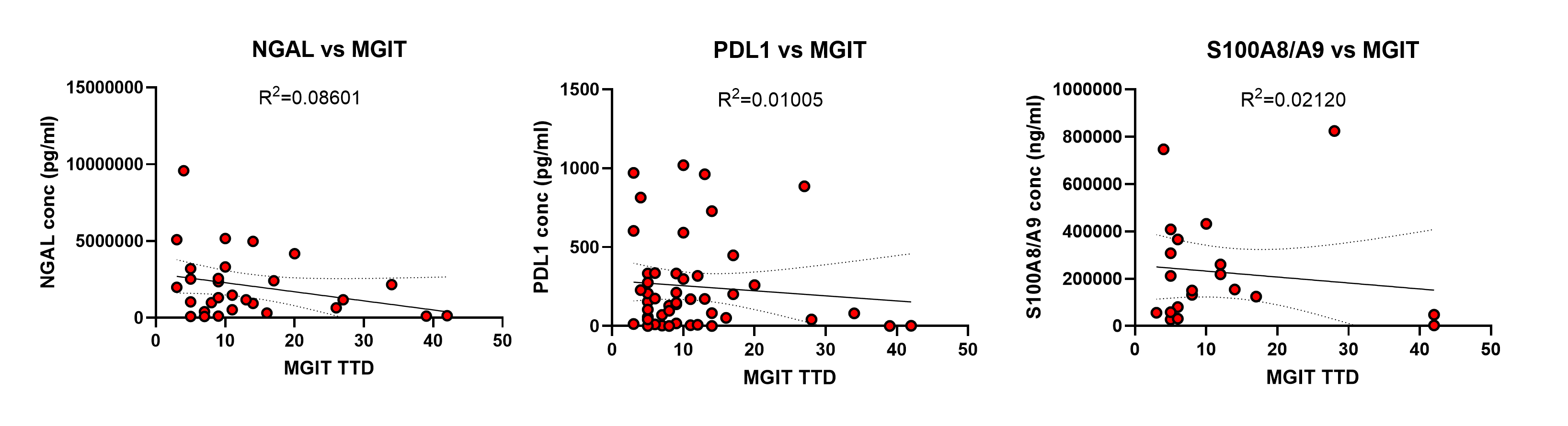

Supplement: Supplementary file 4 [file Image4.tif]

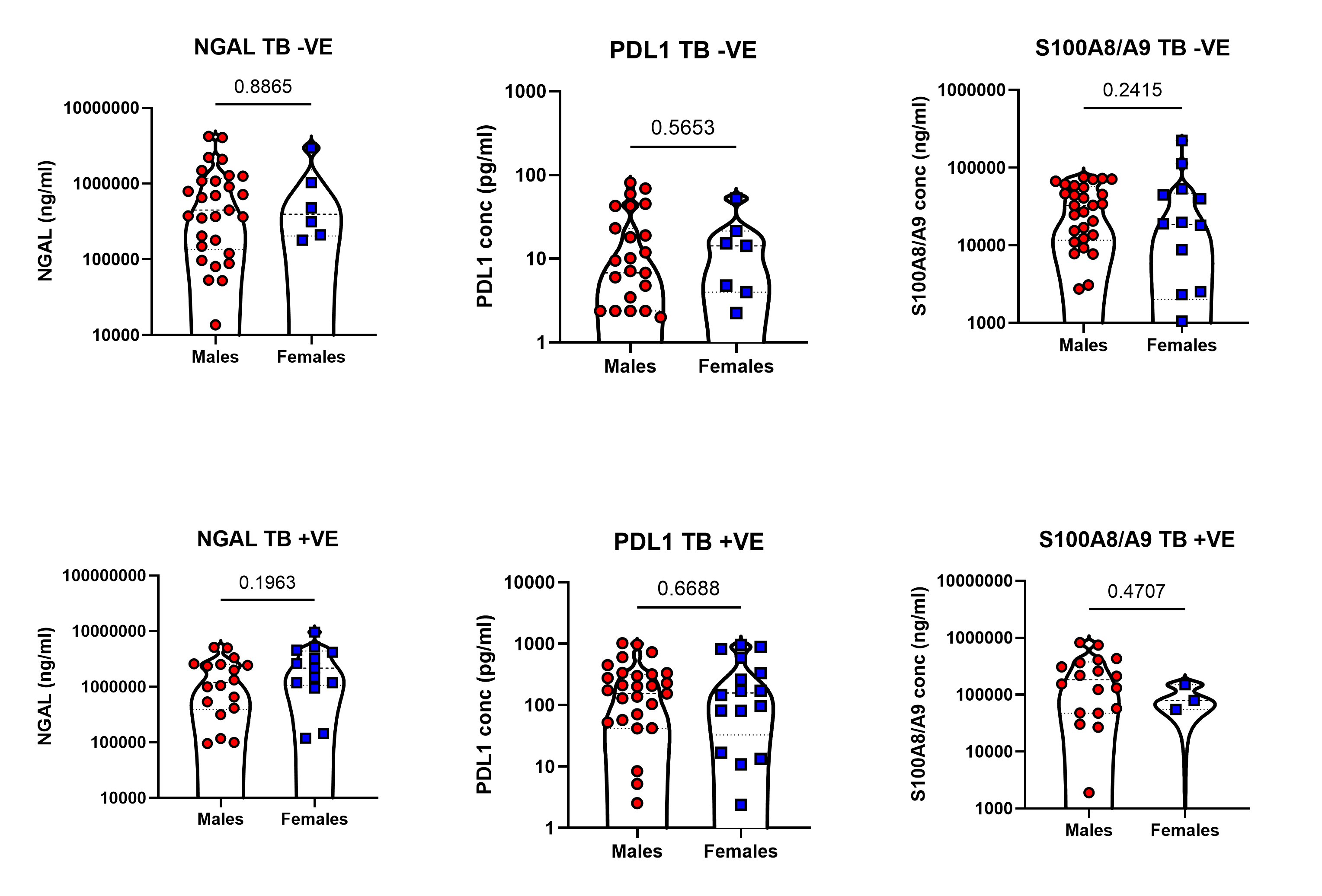

Supplement: Supplementary file 5 [file Image5.tif]

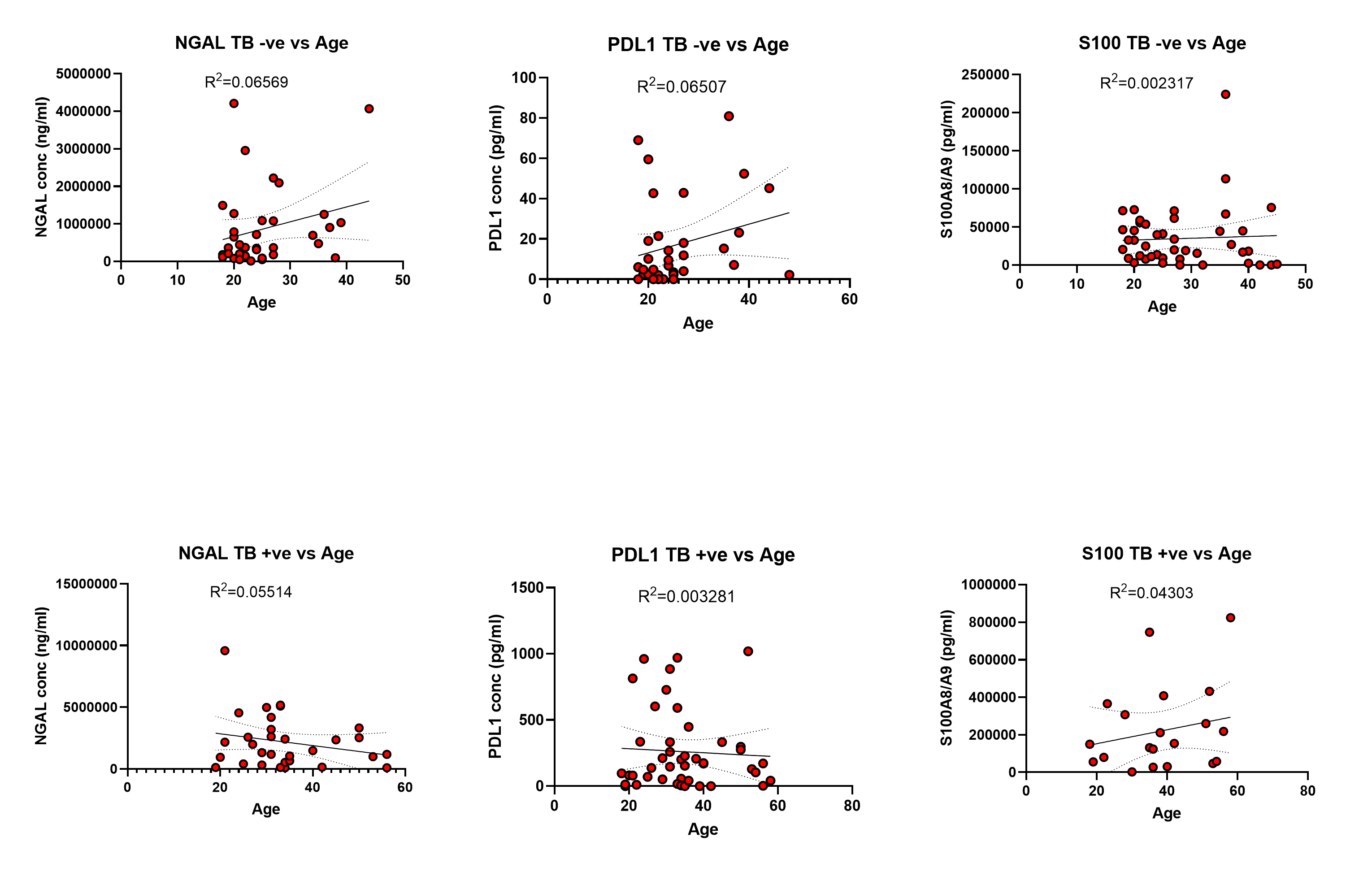

Supplement: Supplementary file 6 [file Image6.tif]

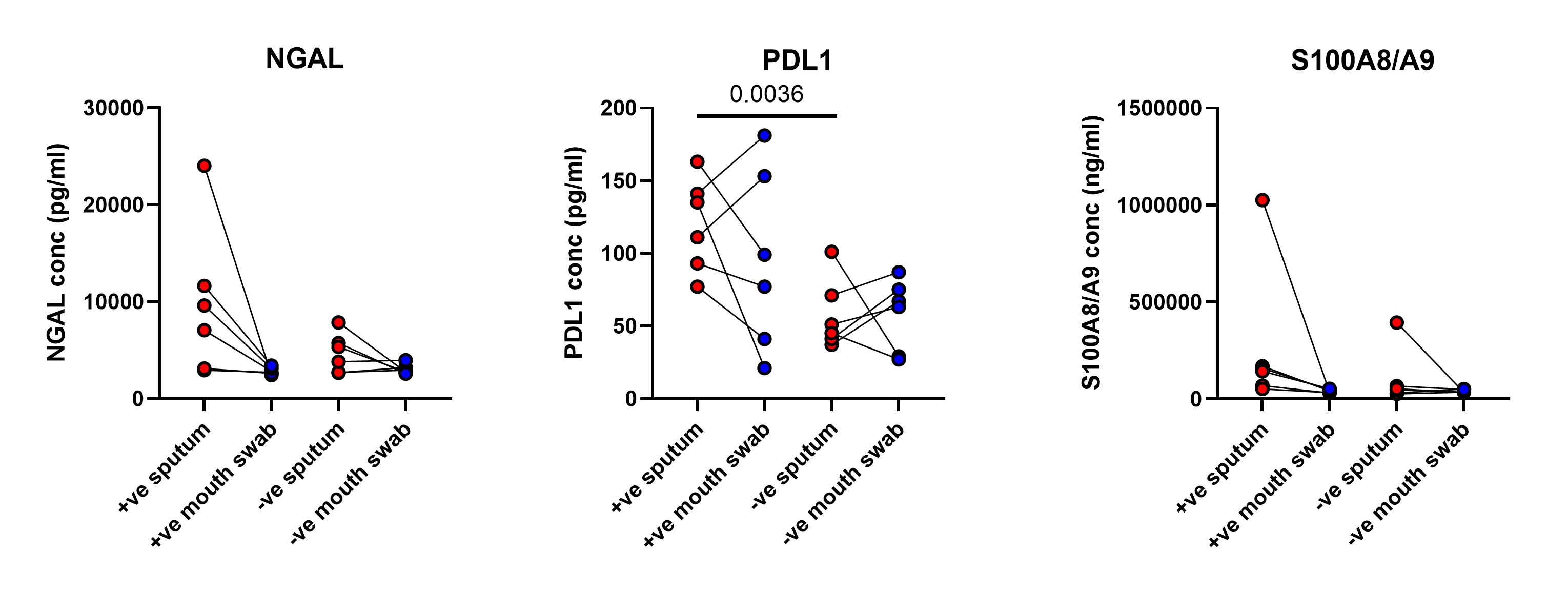

Supplement: Supplementary file 7 [file Image7.tif]

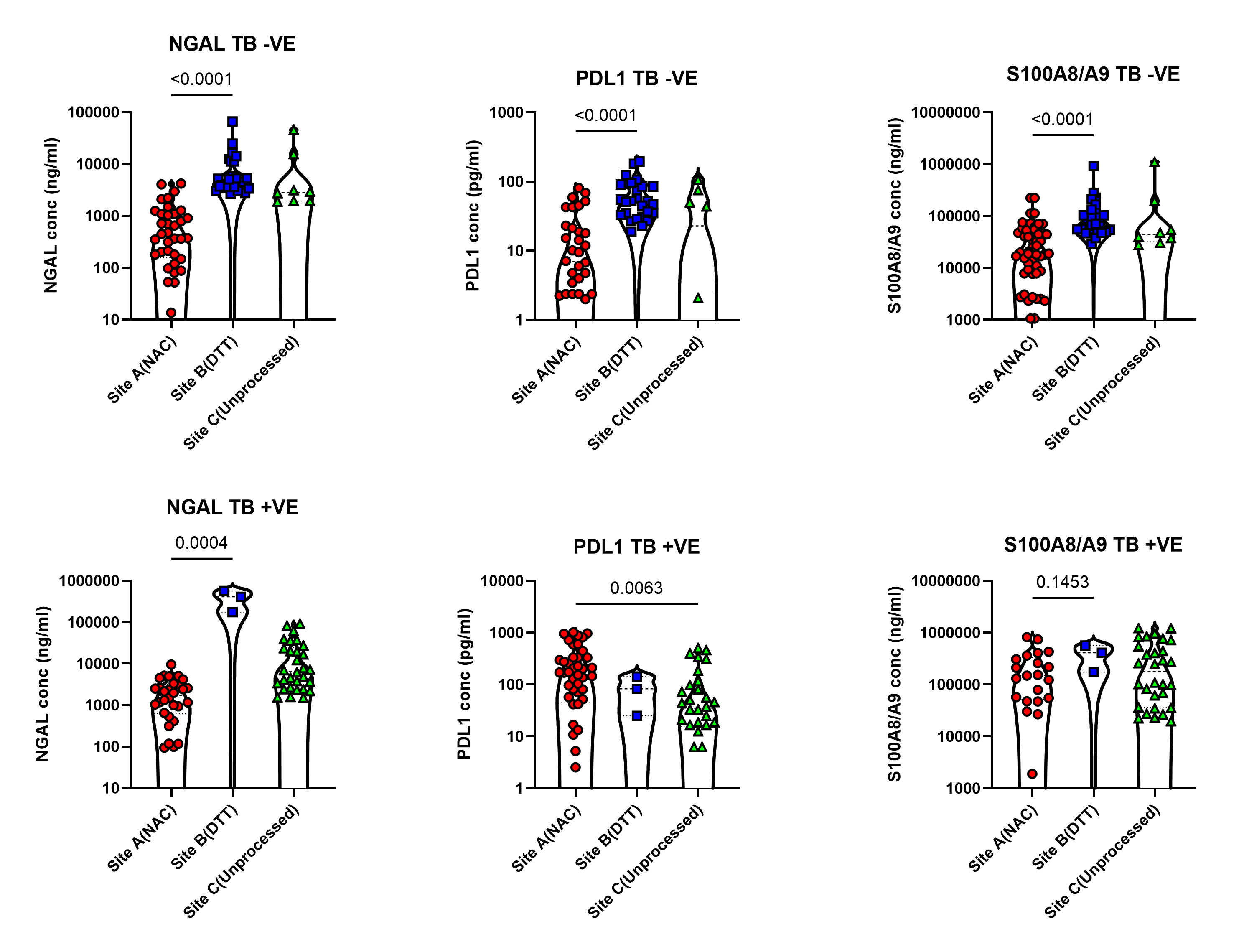

Supplement: Supplementary file 8 [file Image8.tif]
